# Development of a Cure Model for Unsaturated Polyester Resin Systems Based on Processing Conditions

**DOI:** 10.3390/polym16172391

**Published:** 2024-08-23

**Authors:** Abdallah Barakat, Marc Al Ghazal, Romeo Sephyrin Fono Tamo, Akash Phadatare, John Unser, Joshua Hagan, Uday Vaidya

**Affiliations:** 1 Department of Mechanical, Aerospace, and Biomedical Engineering, University of Tennessee, Knoxville, TN 37996, USA; aragab@vols.utk.edu (A.B.); malghaza@vols.utk.edu (M.A.G.); rfonotam@utk.edu (R.S.F.T.); aphadata@vols.utk.edu (A.P.); 2 Composite Applications Group (CAG), 3137 Waterfront Dr, Chattanooga, TN 37419, USA; john@compositeapplicationsgroup.com; 3 Research and Development Department, Wabash National Corporation, 3550 Veterans Memorial Pkwy S, Lafayette, IN 47909, USA; joshua.hagan@onewabash.com; 4 Manufacturing Sciences Division (MSD), Oak Ridge National Laboratory (ORNL), 2350 Cherahala Blvd, Knoxville, TN 37932, USA; 5 The Institute for Advanced Composites Manufacturing Innovation, 2370 Cherahala Blvd, Knoxville, TN 37932, USA

**Keywords:** unsaturated polyester resin, DSC, autocatalytic model, cure kinetics, cure behavior, cure simulation

## Abstract

Unsaturated polyester resin (UPR) systems are extensively used in composite materials for applications in the transportation, marine, and infrastructure sectors. There are continually evolving formulations of UPRs that need to be evaluated and optimized for processing. Differential Scanning Calorimetry (DSC) provides valuable insight into the non-isothermal and isothermal behavior of UPRs within a prescribed temperature range. In the present work, non-isothermal DSC tests were carried out between temperatures of 0.0 °C and 250 °C, through different heating and cooling ramp rates. The isothermal DSC tests were carried out between 0.0 and 170 °C. The instantaneous rate of cure of the tested temperatures were measured. The application of an autocatalytic model in a calculator was used to simulate curing behaviors under different processing conditions. As the temperature increased from 10 °C up to 170 °C, the rate of cure reduced, and the heat of reaction increased. The simulated cure behavior from the DSC data showed that the degree of cure (α) maximum value of 71.25% was achieved at the highest heating temperature of 85 °C. For the low heating temperature, i.e., 5 °C, the maximum degree of cure (α) did not exceed 12% because there was not enough heat to activate the catalyst to crosslink further.

## 1. Introduction

Due to the synthetic nature and liquid phase of polyester, its processing conditions require specific parameters and steps to realize the final product [1,2,3]. These parameters affect the final mechanical and thermal properties of the polyester. Polymerization begins with the raw materials, typically terephthalic acid or its esters, along with ethylene glycol [4,5].

Unsaturated polyester resins (UPRs) are widely used in various sectors such as marine, automotive, and construction infrastructure, to name a few. The chemical process of UPRs includes creating UPRs through poly-esterification or gradual ionic copolymerization. The resulting UPR is dissolved in an unsaturated monomer and subsequently crosslinked using radical polymerization [6,7].

The cure behavior of UPRs is influenced by temperature, catalysts, catalyst concentration, promoters and inhibitors, stoichiometry, resin formulation, atmosphere, curing time, and post-cure processes. These parameters control the transition rate of resin from liquid to solid state through crosslinking. The selection of these parameters is highly dependent on the end-use application and the target properties of the cured material [8,9]. It is also highly dependent on the physical characteristics of the resin system, i.e., resin viscosity, gel point, and vitrification [10].

The resin formulation of UPR influences its curing behavior, which is based on the type of monomers and their functionality [10]. Also, the presence of additives, i.e., reinforcements and fillers, would impact the overall curing process [11,12]. The type of catalysts that initiate the curing reaction, i.e., peroxides, amines, and metal salts, as well as their concentration and amount, significantly influence the curing behavior. These factors affect the desired rate of the curing reaction and the specific properties of the final cured material too [13,14].

Using the proper exact ratio of resin (monomers) and catalyst (crosslinking agents), the optimal curing behavior would be achieved. Moreover, promoters and inhibitors are additives used to control the curing behavior to achieve the desired properties. Promoters and inhibitors are added, if desired, to enhance the activity of catalysts and slow down the curing reaction, respectively.

External parameters also impact the curing behavior, e.g., temperature, pressure, volume, and processing time [15,16]. As the curing reactions are temperature-dependent, heat input provides the necessary activation energy for the reaction [10]. Increasing temperature accelerates curing, while excessive heat degrades the material. Applying pressure to a certain volume of the resin system has a direct effect on its cure behavior. For example, ultra-high curing pressure, around 100–400 MPa, shortens the curing time and reduces the degree of cure and glass transition temperature [17,18]. A significant increase in the curing pressure also might cause a weak glue and fiber distortion, as the resin flows faster in the fiber medium [19,20]. However, decreasing the curing pressure (8–10 MPa, depending on the resin and thickness) allows void accumulation in the matrix [21,22,23,24]. Allowing sufficient time for the curing process achieves the desired mechanical and chemical properties.

Some resin systems require a post-cure stage to enhance the final properties. Environmental conditions also have a critical influence on the curing process. In some cases, the humidity level, the presence of oxygen, and the surrounding temperature influence the curing reactions.

Different studies have optimized the cure profile [25,26,27,28,29,30,31] to minimize the residual stresses and shape distortion, with a reduction in the residual stresses up to 30% [32,33,34,35,36,37]. As the part geometry and thickness influence the cure behavior, it is desired to control the excessive exothermic heat of the thick thermosetting composites. The cure control reduces the process duration for thick parts by 30% [26,27,28], and 50% in the ultra-thick parts [29,30,31]. Several studies have simulated the cure behavior of different resin systems to optimize the cure profile [38,39,40] and to obtain the desired part performance of different processing conditions [41,42,43,44,45,46].

Based on their structure, UPRs are classified as ortho-resins, iso-resins, biphenol-A fumarates, Chlorendics, and vinyl ester resins. Several studies tackled the use of promoters and co-promoters to modify the cure profile of UPRs [47,48,49]. Naderi et al. (2015) [50] added 1–5% nano clay in a UPR containing Na-Montmorillonite to investigate the cure behavior and the cure kinetic parameters. Their investigation showed a decrease in the gel time and exothermic peak, while there was an increase in the cure rate. The inclusion of carbon nanotubes in a polyester resin system (D-EP/CNTs-H20) significantly shifted the cure temperature to a lower temperature while accelerating the cure [51]. This shows the cure reaction can be accelerated at a lower temperature provided the relevant catalyst is used. Calabrese et al. (2023) [52] demonstrated that commercial unsaturated polyester imide resins possessed excellent thermal resistance of up to 320 °C.

An examination of the effect of cure temperature on the mechanical properties of a polyester resin was carried out by Silva et al. (2020) [15]. Their results indicated a significant improvement of the bending strength when the resin was post-cured at 40 °C and 60 °C as compared to room temperature. Nacher et al. (2007) [53] examined how the curing rate influences the mechanical properties of a polyester resin immersed in saline water for 500 h. They realized that the highly crosslinked internal structure of the resin presents a smaller capacity to absorb water than a structure with a low degree of crosslinking. Moreover, examination of the influence of curing rate on the mechanical properties of polyester resin under saline water revealed that the highly crosslinked resin structure withstands more compared to the resin system with a low degree of crosslinking due to lower water absorption [53]. Furthermore, an important increase in tensile strength was observed with the crosslinking grade for each cured temperature. For instance, at the cured temperature of 60 °C, tensile strength increased from 14.6 MPa for 60% of crosslinking to 34.1 MPa for 90% in reference samples. But in the degradation samples, this increase was from 11.3 to 28.2 MPa.

The glass transition phase is an important factor in the cure condition of unsaturated polyester resins. There has been keen interest among researchers to increase the glass transition temperature without altering the crosslink of the UPRs. By raising the styrene content from 35 to 50 wt% in their newly developed thermal curing profile without thermal initiators, Stuck et al. (2020) [54] saw a drastic increase in the glass transition temperature (195–215 °C) at 10 Hz. Delaite et al. (2020) [55] enhanced the glass transition temperature ($T_{g}$) from 197 °C to 130 °C by replacing 35 wt% of styrene with butyl methacrylate.

The degradation of unsaturated polyester resin depends mostly on the environmental conditions, and it can take various courses, as evaluated by Pączkowski et al. (2020) [56]. UPR is subjected to accelerated aging, immersion in different solvents, and high temperatures for the purpose of comparing their degradation.

There are many processing parameters involved during resin curing; therefore, extensive testing and optimization are needed for new UPR formulations. The main objective of this work was to create a tool to determine the optimal process conditions for the UPR system. Material characterizations were conducted to evaluate the conversion rate of the resin system with different processing parameters. A model was developed to simulate the curing process based on the desired input processing parameters. The study aims to employ an approach to comprehend the behavior of resin systems under any given processing condition through a systematic solution to optimize processing conditions. The objectives of the present work are summarized as follows:

Conducting gel tests and TGA and DSC runs to characterize and study the cure kinetics of two resin systems.

Simulating the cure kinetics behaviors under different temperatures for the two resin systems using the autocatalytic model.

## 2. Experimental and Numerical Methodology

A material characterization study was carried out on two UPR systems through various techniques including the gelation test, thermographic analysis (TGA), and DSC. These two resin systems are referred to as Resin system 1 (COR61-AA-248S) and Resin system 2 (COR61-AA-270LF).

### 2.1. Polymer Characterization

Three gel tests were conducted on each UPR system 1 and 2. Temperature was recorded throughout the curing process by the HH374-Omega data logger thermometer and Omega thermocouples. For each test, 100 g of resin was mixed with 1.0 g (1.0 wt%) of Norox methyl ethyl ketone peroxide (MEKP)-925H initiator. The mixture was placed in an open polypropylene container made by FibreGlast, with a thermal conductivity coefficient of 0.2 W/(m°∁).

The TGA Q50 (V20.13, Build 39) instrument was used to understand the mass loss of both polyester resins. Around 15.0 mg of resin was placed in a platinum pan. The sample chamber was flushed with nitrogen. The ramp rate was 10 °C/min up to a maximum temperature of 500 °C. The resin degradation temperature was recorded.

TA Instruments DSC Q-2000 was used for the measurement of evolved heat during the cure reaction of UPR with 1.0% MEKP. All the DSC experiments were carried out using hermetic aluminum sample pans with sample weights of 20 mg (±4 mg). The isothermal reaction rate versus time profiles were measured at 10–120 °C and 170 °C. The isothermal runs were conducted as per Table 1. Isothermal DSC runs with no initiator were performed for 10 min to evaluate the impact of heat on the resin.

The total heat of the cure reaction (HU) was investigated using the measurement of heat flow with heating ramps for 5 °C/min, 10 °C/min, and 20 °C/min using dynamic DSC experiments carried out from temperature range 0 to 250 °C. For non-isothermal DSC runs, Tert-butyl peroxybenzoate (Trigonox C) was used as an initiator due to its thermal stability at high temperature. The following equations were used for the heat of reaction calculation [57,58,59,60].

The total heat of the reaction (HU) can be computed by the non-isothermal DSC runs. Equation (1) is used for HU calculation:

(1) $$HU=\int_{0}^{td}\left(\frac{dQ}{dt}\right)d\;dt$$

where $\left(\frac{dQ}{dt}\right)d$ represents the instantaneous rate of heat produced, while *td* corresponds to the time required for the completion of the reaction in the non-isothermal DSC experiment.

Equation (2) gives the amount of energy (heat generation) in an isothermal experiment form the start of the cure up to the time t:

(2) $$H(t)=\int_{0}^{t}\;\left(\frac{dQ}{dt}\right)T\;dt$$

where $\left(\frac{dQ}{dt}\right)T$ represents the instantaneous rate of energy generated at temperature T.

The heat generation H(t) is directly proportional to the degree of reaction, $\alpha \left(t\right)$, characterized by Equation (3):

(3) $$\alpha \left(t\right)=\frac{H(t)}{HU}$$

The total isothermal heat of reaction (HT) from isothermal scanning experiments can be expressed by Equation (4):

(4) $$HT=\int_{0}^{ti}\left(\frac{dQ}{dt}\right)i\;dt$$

Similarly, in case of isothermal scanning runs, $\left(\frac{dQ}{dt}\right)i$ represents the instantaneous rate of heat generated and ti is the amount of time required to complete the reaction.

The actual rate of cure as function of time $\left(\frac{d\alpha }{dt}\right)$ and the isothermal rate of cure $\left(\frac{d\beta }{dt}\right)$ can be defined by Equations (5) and (6) [60]:

(5) $$\frac{d\alpha }{dt}=\frac{1}{HU}\;\left(\frac{dQ}{dt}\right)T$$

(6) $$\frac{d\beta }{dt}=\frac{1}{HT}\;\left(\frac{dQ}{dt}\right)T$$

Furthermore, the actual rate of cure $\left(\frac{d\alpha }{dt}\right)$ can be related to the isothermal rate of cure $\left(\frac{d\beta }{dt}\right)$, as per Equation (7):

(7) $$\frac{d\alpha }{dt}=\frac{HT}{HU}\;\frac{1}{HT}\;\left(\frac{dQ}{dt}\right)T=\frac{HT}{HU}\;\frac{d\beta }{dt}$$

β(t) is defined as the extent of reaction in the isothermal scan at time t which can be expressed by Equation (8):

(8) $$\beta \left(t\right)=\frac{H(t)}{HT}$$

The ratio between HT and HU expresses the degree of incomplete reaction and can be thought of as a piecewise function, as shown in Equation (9):

(9) $$\frac{HT}{HU}=\left\{\begin{array}{cc} f\left(T\right) & T<Tc\\ 1 & T\geq Tc\; \end{array}\right.$$

For a given heating time, and above some critical temperature $T_{c}$, the isothermal cure will reach complete reaction.

The process of studying cure kinetics using DSC involves a crucial step where the obtained reaction rate profile from experiments is fitted to a kinetic model. These models can be categorized as either phenomenological or mechanistic [61]. In phenomenological models, a relatively simple equation is utilized, disregarding the intricate details of the specifics of reactive species involvement in the reaction. On the other hand, mechanistic models are developed from balances of the reactive species participating in the reaction. Although mechanistic models provide more accurate predictions, deriving them is often not feasible due to the complex nature of cure reactions. In this study, the resin system exhibited the autocatalytic effects defined by Equation (10) [62].

(10) $$\frac{d\alpha }{dt}=k\alpha^{m}{\left(1-\alpha \right)}^{n}$$

where k represents the reaction constant defined by the Arrhenius law $\left(k\left[T\right]={Ae}^{\frac{-E}{RT}}\right)$, T is the absolute temperature, R is the universal gas constant, A is the pre-exponential factor, E is the activation energy, and m and n are the reaction orders. The rate coefficients and reaction order values are calculated and used as proprietary information.

### 2.2. Cure Simulation

A numerical simulation was used to simulate the cure behavior of different processing conditions. The temperature variation of the heat input and the surrounding area are critical to control the cure behavior. Predicting correct cure behavior at each temperature through simulations is essential. However, testing all the possible temperatures is not feasible through DSC.

**Figure 1:**
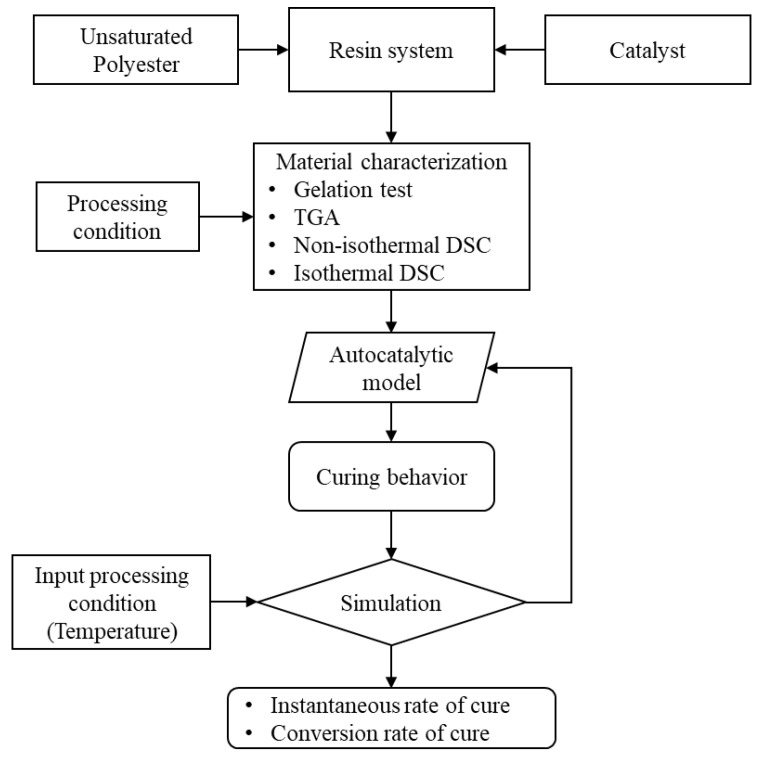
A flow diagram illustrates the simulation process of the cure behavior. The autocatalytic model utilizes the heat of reaction of the resin system and yields the rate of cure and conversion rate. These values vary based on the input processing temperature, which in turn influences the heat of reaction.

In Figure 1, a schematic shows the analysis steps and the simulation sequence. The material characterization process generates the data to evaluate the curing behavior of the UPR system. The evaluation of those data were conducted through the autocatalytic model, as mentioned previously. There is a relation between the rate of cure, degree of cure, curing temperature, and curing time [63,64,65,66], as shown in Equation (1) to Equation (9). This relation accurately predicts the cure behavior [65]. Also, it could be used to capture the effect of the reinforcing materials on the curing kinetics [67,68,69,70,71,72]. An iterative process fits the DSC data and evaluates the corresponding activation energy of the resin system. The activation energy governs the reaction behavior, speed, and the exothermic heat. The process also evaluates some main values based on the autocatalytic model equations.

The input parameters were introduced, and an interpolation process calculates the results corresponding to the desired value. For example, the tested DSC for a certain temperature is already existing; however, a fraction of the temperature up or down is not available. Therefore, the simulation finds that results and evaluates the cure behavior accordingly. The model interpolates between each time step to find the degree of conversion corresponding to the exact temperature. The unknown degree of conversion was evaluated though an interpolation process.

As the used catalyst type is heat activated, mainly the heat input represented in the heating temperature is essential. The used temperature would be used as a constant temperature, or even a variable temperature. In the case of a variable temperature, the controlled temperature is divided into three main zones: first the heating zone with a ramp rate, dwell time with a constant temperature, then a cooling temperature with a ramp rate.

A MATLAB code was developed with a graphical user interface (GUI) to process these inputs through the autocatalytic model using MATLAB R2023b. The code uses a limited number of DSC data to predict the cure behavior of the resin system. Moreover, the model evaluates the heat of reaction, heat output, and degree of cure (α). 

## 3. Results and Discussion

### 3.1. Characterization Results

**Figure 2:**
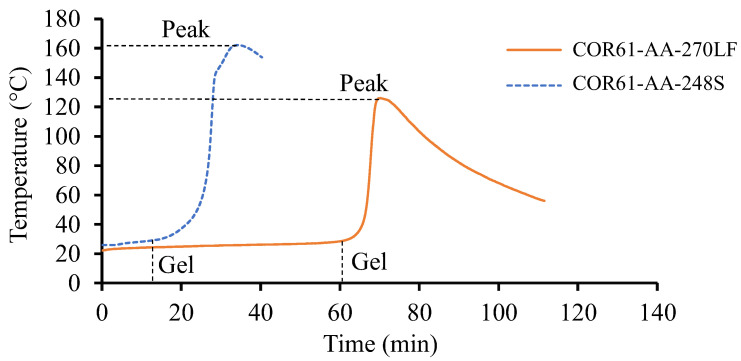
Gel test results for Resin 1 and Resin 2. The test was conducted for a sufficient period until the peak temperature was achieved and no further heat release was recorded. Resin 2, as a fast-cure system, exhibits a higher release rate and provides a greater exothermic heat.

No visible separation between the samples and respective containers was observed during the gel tests. The average temperature against time plots were concluded on both resin systems and are shown in Figure 2. The initial temperature of the resins was dependent on the room temperature. COR61-AA-248S started at 26 °C, while the initial temperature of COR61-AA-270LF was 22 °C. There are significant differences between the two curves. COR61-AA-248S started transitioning from a liquid to gel state after 13 min. For COR61-AA-270LF, the reaction started after 60 min, a 361.5% longer time. The peak exotherms were also different; COR61-AA-248S reached its highest temperature of 162 °C after 34 min, while COR61-AA-270LF exhibited a 20% lower peak (126 °C) after 70 min, a 105.9% longer time. The difference in behaviors between the two polyester resins was caused by their distinct chemistries and crosslinking networks. The gelation results were compared to the available literature [73,74].

The peak exotherms obtained in this research (126 °C and 162 °C) compared well with the literature values (120 °C and 150 °C). The higher values (162 °C and 350 °C) had only a 7.3% difference and the lower ones (126 °C and 120 °C) had an even smaller difference of 4.4%. The gel times were harder to compare because of the effects of accelerators mixed with the UPR/MEKP system, but they served as indicators for the starting times of the cure reactions.

**Figure 3:**
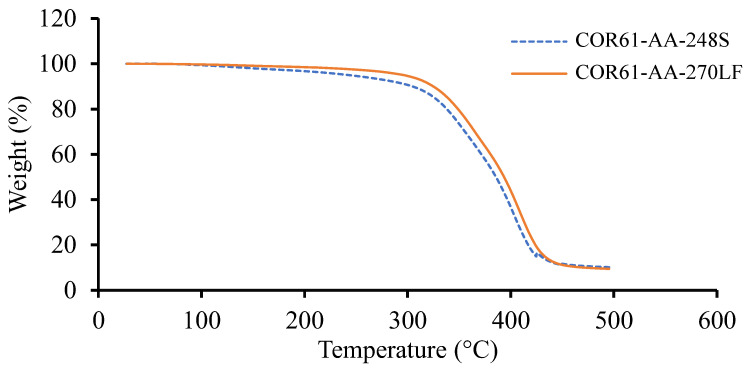
TGA results for Resin 1 and Resin 2. Both resin systems exhibited similar trends; however, COR61-AA-248S begins to lose weight more rapidly than COR61-AA-270LF. The decomposition of Resin 1 and Resin 2 at $T_{5}$ occurs at 243 °C and 296 °C, respectively.

Based on the TGA results, the weight change for the corresponding temperature plots for Resin 1 and Resin 2 are shown in Figure 3. Both resins experienced a single stage decomposition. The temperatures at 10 wt% weight loss ($T_{10}$) and the maximum temperatures ($T_{max}$) are given in Table 2 for both UPRs. Similar results were observed for both resins; Resin 1 had a $T_{5}$ of 243.03 °C, $T_{10}$ of 304.64 °C, and a $T_{max}$ of 416.88 °C, while Resin 2 gave higher values of $T_{5}$ of 296.44 °C (22% higher), $T_{10}$ of 324.87 °C (6.6% higher), and a $T_{max}$ of 423.84 °C (1.7% higher). To compare the results of this study with other research, the literature was reviewed.

Dai et al. [75] experimented on a UPR and obtained a $T_{10}$ of 348 °C and $T_{max}$ of 419 °C. Pączkowski et al. [56] studied the properties of an orthophthalic-based UPR and observed a $T_{10}$ of 336 °C and $T_{max}$ of 394 °C. Similarly, Tibiletti et al. [76] obtained a $T_{5}$ of 287 °C and $T_{max}$ of 427 °C for their orthophthalic-based UPR. Finally, Bai et al. [77] also performed TGA on an orthophthalic-based UPR and obtained resulting temperatures of $T_{5}$ equal to 307 °C and $T_{max}$ equal to 433 °C. All the literature results are given in Table 3. The results of Resin 1 and Resin 2 are comparable to the literature values. The TGA results were used to ensure that no temperature values exceeded the initial degradation temperatures ($T_{5}$) while conducting DSC.

**Figure 4:**
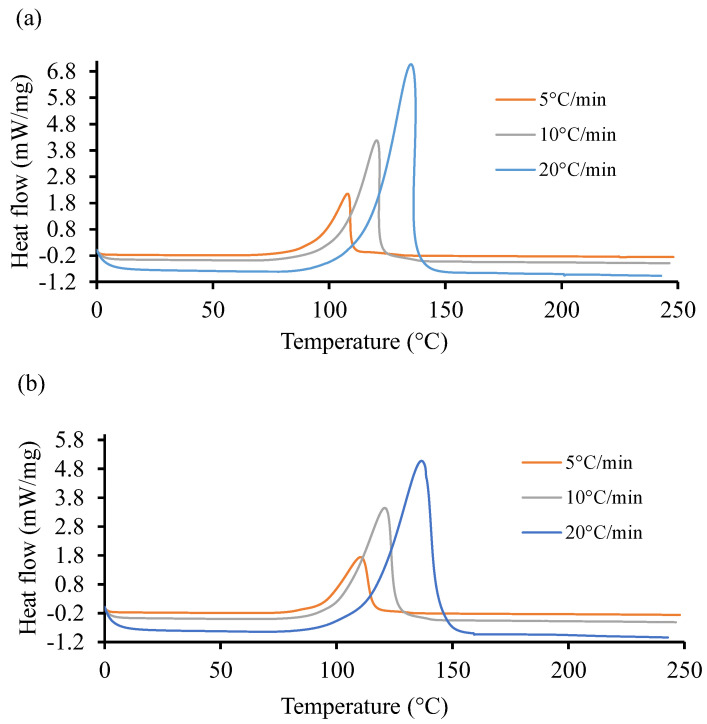
Heat flow of resin systems corresponding to three heating ramp rates, 5, 10, and 20 °C. (**a**) Non-isothermal DSC runs for Resin 1 and (**b**) non-isothermal DSC runs for Resin 2.

Figure 4 shows the non-isothermal DSC curves of the UPR for the two resin systems at three different heating ramps of 5, 10, and 20 °C. It is evident in these graphs that the cure reactions took place in one stage, regardless of the heating rate for both resin systems. Both the initiation temperature and final temperature increased with an increase in scan rates, similar to the observation reported by Sultania et al. (2012) [78]. An estimation of the area under the curve of the high heating rate is higher than in the other two cases. This behavior could be explained by a complete curing at a higher heating rate. In Figure 4a, the heat flow rate is substantially higher (6.8 mW/mg) as compared to the same in Figure 4b. The peak exothermic temperature for the highest heat flow rate is approximately the same in both cases and situated around 145 °C.

The total heat of reaction (HU) was computed around 341.62 J/g with 13.88 J/g standard deviation (S.D.) for Resin 1. Similarly, for Resin 2, the HU was measured about 365.8 J/g with an S.D. of 11.37 J/g. Furthermore, the analysis of non-isothermal and isothermal DSC data resulted in the parametric constants of the autocatalytic model using a weighted least squares non-linear regression method with a 95% confidence interval [79]. The obtained parameters could not be revealed due to sensitivity of the project outcome. Unlike the work of Gohn et al. (2019) [80], increasing cooling rate tends to suppress the cure temperature to lower temperatures. Meanwhile, Figure 4, displaying the non-isothermal cure curves, shows that increasing the ramp rate pushes the cure temperatures to higher values.

At the lowest rate, 5 °C/min, Resin system 1 has a peak cure temperature ($T_{c}$) of 110 °C and Resin 2 has a $T_{c}$ of 115.9 °C, resulting in a 5.3 °C difference. At the maximum rate, 20 °C/min, Resin 1 has a $T_{c}$ of 146.3 °C, while Resin 2 has a $T_{c}$ of 148.1 °C, resulting in a 2.0 °C difference. It is apparent that the Resin 2 additive has a strong effect on non-isothermal cure at low cooling rates but is more pronounced at higher rates.

**Figure 5:**
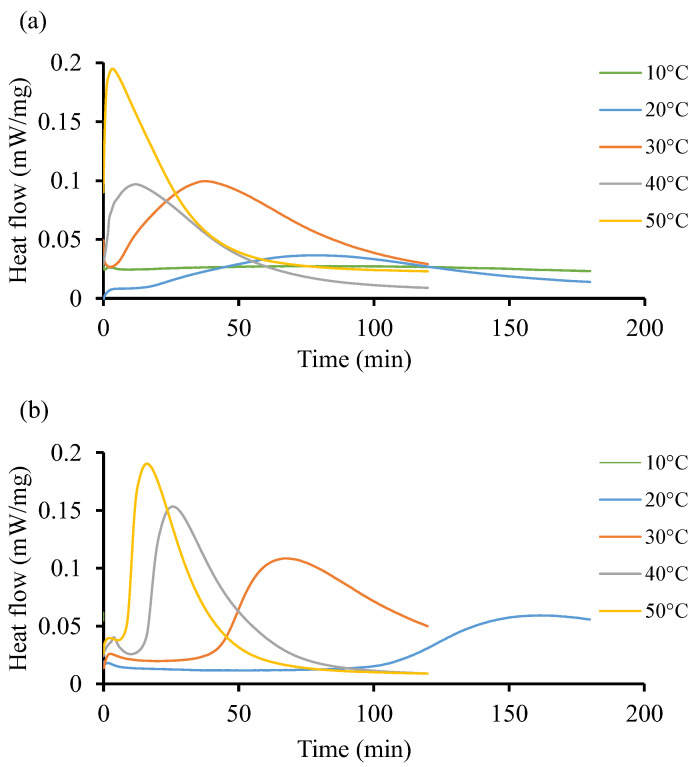
Heat flow of resin systems corresponding to temperature range from 10 °C to 50 °C without initiator. (**a**) Isothermal DSC runs for Resin 1 and (**b**) isothermal DSC runs for Resin 2.

Figure 5 shows the isothermal reaction of the two resin systems at low temperature, from 10 °C to 50 °C. Both resin systems behave differently without following a particular pattern. For instance, Figure 5a shows that at 10 °C, there is no significant impact on the curing behavior of the resin. It takes up to 79 min to record a low heat flow (0.027 mW/mg). In Figure 5b, the initiation process is faster as it occurs in less than a minute at the start of the test and the heat flow remains low (0.062 mW/mg), although this value is higher than that recorded in Resin 1. At the isothermal temperature of 50 °C, both resin systems achieve relatively similar heat flow patterns of 0.195 and 0.190 mW/mg, respectively, but at a shorter time for Resin 1 (3 min) as compared to Resin 2 (16 min). Furthermore, Figure 5b shows heat flow peaks at 30 and 40 °C only. The curves are flat throughout at 10, 20, and 50 °C. Such instability could be explained by the nature of the initiators used in both resin systems, whereby the crosslinking of the polymeric chains does not follow a pattern. Due to the large area under the curves in Figure 5b, it can be determined that the total heat released during the isothermal cycle is considerable compared to the same area in Figure 5a.

**Figure 6:**
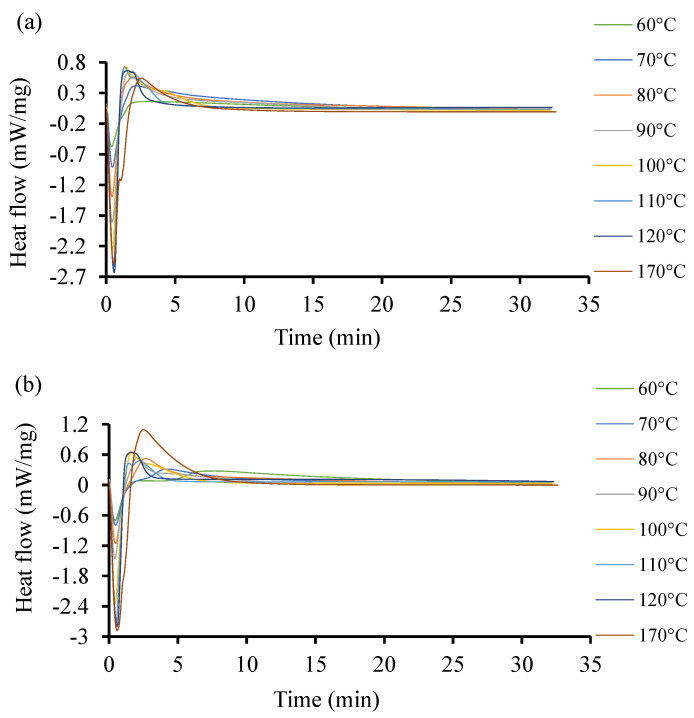
Heat flow of resin systems corresponding to temperature range from 60 °C to 170 °C with initiator. (**a**) Isothermal DSC runs for Resin 1 and (**b**) isothermal DSC runs for Resin 2.

To further investigate the discrepancies observed in the heat flow of the two resin systems at low temperatures with initiators, more DSC experiments were conducted at higher temperatures between 60 and 170 °C. The acquired curves are shown in Figure 6. The curves exhibit different induction times. Unlike in the previous case at low temperature, the heat flow here increases as the temperature increases. The highest heat flow corresponds to the highest temperature in both cases. Resin 2, as shown Figure 6b, can achieve a higher temperature and higher heat flow rate as compared to Resin 1. The optimal interval is not easily identifiable as the induction of reactions is around 1 min. One distinctive observation here is that at higher temperatures, the curing time is lower in both resin systems. This same trend was observed by Salla and Ramis (1996) [59] while using different procedures to study the cure kinetics of a UPR.

In Figure 6, both resin systems were subjected to a high temperature isothermal cure ranging between 10 and 170 °C. It is shown that the isothermal cure of Resin 1 could only be quantified up to temperatures of 120 °C. At higher temperatures, the cure rate was too slow to produce a detectable calorimetric signal. However, Resin 2 was able to crosslink at temperatures up to 170 °C. A similar trend was deduced by Gohn et al. (2019) [80] in their study on understanding the cure kinetics in two different composites.

**Figure 7:**
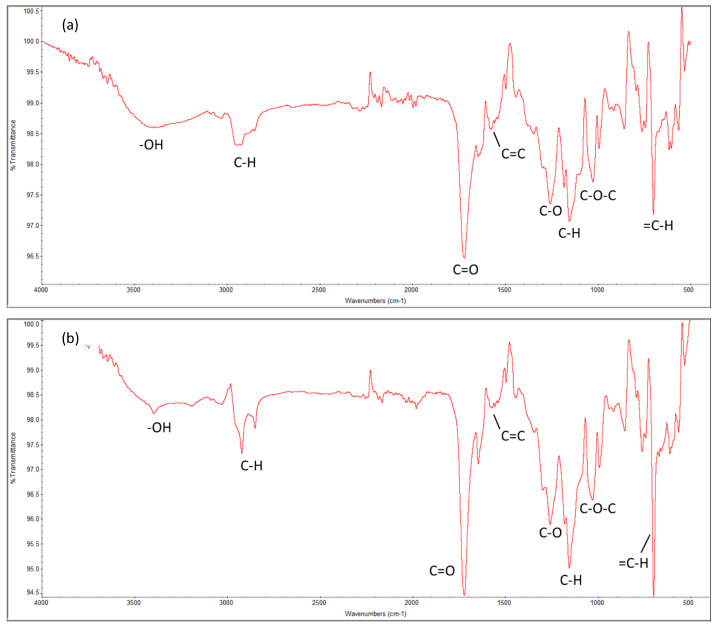
Fourier-transform infrared (FTIR) spectrum of the cured UPRs for both resin systems. (**a**) Resin 1 and (**b**) Resin 2.

The molecular-level information determines the behavior of the systems. The chemical properties of the cured resin system at 70 °C of both Resin 1 and Resin 2, as characterized by Fourier-transform infrared spectroscopy (FTIR), are shown in Figure 7. It highlights the main functional groups of UPR, i.e., the polyester linkages of carbon–oxygen bonds (C-O-C, C=O and C-O), aromatic hydrocarbons of styrene (C-H), and the hydroxyl (OH) groups. These results match with data from the literature [81].

### 3.2. Cure Behavior

The heat of reaction from the DSC represents the endothermic and exothermic behavior of the reaction but does not represent the conversion rate. The autocatalytic curing model was used to evaluate the degree of cure (α). The non-isothermal DSC data were used to evaluate the total heat of reaction (HU), which was used to represent the heat behavior of the resin system under different conditions. The glass transition temperature ($T_{g}$) was specified from the peak value of the reaction, as shown in Figure 4.

The non-isothermal tests were carried out between temperatures of 0.0 °C and 250 °C, through different heating and cooling ramp rates. It ensures the full cure of the samples, which determines the exact heat of reaction of the resin system. It was noticed that there was no peak in the second heat cycle, which indicates that it was difficult to further break the crosslinking, as the polymeric chains could not vibrate due to heat. The heat of reaction of the resin system is given by the heat capacity value.

As shown in Figure 6, the DSC results are limited to the tested temperatures, e.g., 60.0 °C, 120.0 °C, etc. But, as the applied temperature varies due to internal or external factors, the cure behavior changes accordingly. Implementing all the possible cases of DSC testing could be an extensive process. Therefore, the simulation of the cure behavior solves this problem. To obtain the cure behavior correctly, the rate of cure was captured to identify the reaction speed and intensity. The instantaneous rate of cure was measured based on Equation (5).

The heat input for the resin system is the main contributor to activate the catalyst and the crosslinking reaction. The amount of added catalyst defines the total degree of cure, which is based on the total activation energy. Hence, the pressure applied, volume of the system, and the temperature applied through the PVT method dictate the cure behavior, cure rate, and cure amount [82]. Controlling that temperature is essential, and mainly the temperature was used as a constant temperature or a variable temperature. The definition of the temperature profile depends on the resin system, the cycle time, and the end use application. The simulation functionality finds the degree of cure of any given constant or variable temperatures in between the tested values to optimize the heating cycle. The variable heating temperature was used in several end-use applications, e.g., autoclave, pultrusion, etc.

**Figure 8:**
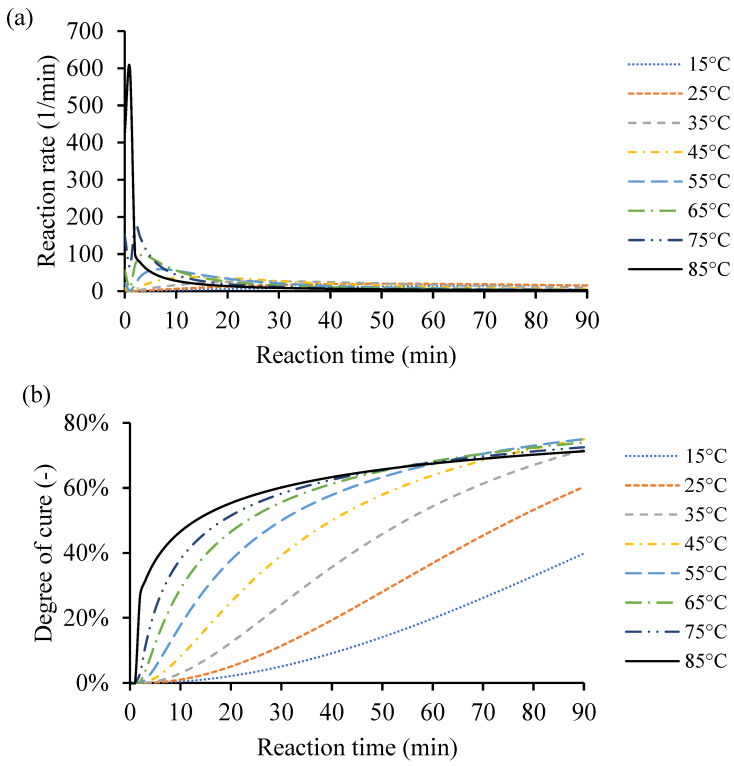
An example of the cure kinetics behavior of Resin system 1 simulated by the MATLAB GUI using constant processing temperatures of 15 °C, 25 °C, 35 °C, 45 °C, 55 °C, 65 °C, 75 °C, and 85 °C. (**a**) Instantaneous rate of cure corresponding to reaction time. (**b**) Degree of cure over time.

As the temperature increases from 10 °C up to 170 °C, the rate of cure reduces, and the heat of reaction increases. The amount of heat released from the different samples is close to each other. However, the external heating temperature defines the distribution and heat release from the exothermic reaction. Also, the amount of catalyst in the end-use application affects the heat release distribution, as it impacts the crosslinking process and, consequently, the amount of heat due to the reaction. Figure 8a shows an example of the instantaneous rate of cure $\left(\frac{dQ}{dt}\right)$ calculated based on constant processing temperatures of 15 °C, 25 °C, 35 °C, 45 °C, 55 °C, 65 °C, 75 °C, and 85 °C.

Based on the raw DSC data, the cure behavior was simulated from the same temperatures used in that case study (10–170 °C). For the simulated low heating temperature, i.e., 5 °C, the maximum degree of cure (α) did not exceed 12% because there was not enough heat to activate the catalyst to crosslink more. The high heating temperature evaporates the remaining monomers in the resin system, which limits the resin system to have a higher conversion value. Finding the proper heating input for the resin system is essential to obtain the desired conversion rate in time. Thus, the highest heating temperature was limited to 85 °C and achieved degree of cure (α) value of 71.25%, to avoid material vitrification. The type and amount of catalyst dictates the cure behaviors. Any excess heat input leads to vitrification of the resin system, and then degradation of the system. Figure 8b shows the corresponding degrees of cure (α) of the constant heating temperatures 15 °C, 25 °C, 35 °C, 45 °C, 55 °C, 65 °C, 75 °C, and 85 °C during 90 min. The degree of cure was increased by increasing the processing temperature to the maximum of 85 °C. Any given processing temperature within that range for any of the processed resin systems would be simulated through the MATLAB GUI. The cure behavior at this point is assumed to simulate a very thin plate. The kinetic equations are necessary; however, the thermal conductivity coefficient and heat transfer equations are neglected for simplicity.

**Figure 9:**
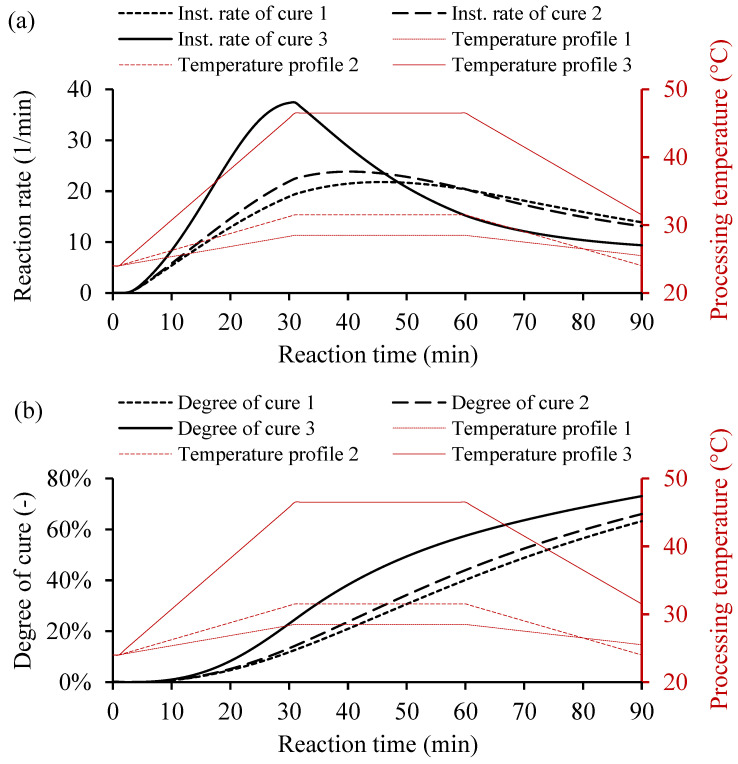
Examples of cure kinetics behaviors of Resin system 1 simulated by MATLAB GUI using variable heating temperature. (**a**) Corresponding instantaneous rate of cure. (**b**) Degree of cure for a variable processing temperature, including heating up with a ramp rate, dwell time at a constant temperature, and cooling down with a ramp rate.

For the variable heating temperature, the curing cycle would be controlled as desired. Usually, the heat cycle consists of three main heating zones, i.e., the heating up, dwelling, and cooling down. Each heat zone has a function that affects the curing behavior of the resin system. The heating and the cooling cycles use a ramp rate to avoid any thermal stresses on the cooling and the rapid curing, which causes undesired shrinkage of the part. However, the dwell zone provides sufficient heat to cure the resin volume. Figure 9 shows three temperature profiles and the yielded instantaneous rate of cure and degree of cure. The variable heating temperatures consists of three main cycles, heating up with a ramp rate, dwell time for consistent temperature, and cooling down with a ramp rate, as shown in Table 4.

In Figure 9a, the instantaneous rate of cure of Resin 1 based on variable heating temperatures shows the heat distribution over time. The high heating temperature, as in case 3, increases the reaction rate rapidly unlike the lower heating temperatures. However, the conversion rate behaves in a different fashion than the constant heating temperatures, as shown in Figure 9b. The increased temperature did not increase the degree of cure significantly, which could save energy during manufacturing. The calculated degrees of cure for 90 min were 63.19%, 66.05%, and 73.02% for cases 1, 2, and 3, respectively (see Table 4). The instantaneous rate of cure controls the cure cycle, which depends on the variable thermal cycle to reach the desired amount of cure. Thus, the curing behavior of the resin system could be controlled and adapted for different manufacturing processes under varying processing conditions.

## 4. Summary and Conclusions

In this work, an autocatalytic model was used to develop a calculator that can accurately provide cure profiles for different UPRs considering the processing conditions. The initial characterization of the UPRs involved the gel time, the TGA, the non-isothermal, and the isothermal DSC tests. The following were observed:

- For the gel time, COR61-AA-248S started transitioning from a liquid to gel state after 13 min, while the COR61-AA-270LF reaction started after 60 min, making it a 361.5% longer time. COR61-AA-248S reached its highest temperature of 162 °C after 34 min, while COR61-AA-270LF exhibited a 20% lower peak (126 °C) after 70 min, a 105.9% longer time. The difference in behaviors between the two polyester resins was caused by their distinct chemistries and crosslinking networks.
- The TGA showed that Resin 1 had a $T_{5}$ of 243.03 °C, $T_{10}$ of 304.64 °C, and a $T_{max}$ of 416.88 °C, while Resin 2 gave higher values of $T_{5}$ of 296.44 °C (22% higher), $T_{10}$ of 324.87 °C (6.6% higher), and a $T_{max}$ of 423.84 °C (1.7% higher).
- At the lowest rate of 5 °C/min, Resin system 1 had a peak cure temperature ($T_{c}$) of 110 °C, and Resin 2 had a $T_{c}$ of 115.9 °C, resulting in a 5.3 °C difference. At the maximum rate of 20 °C/min, Resin 1 had a $T_{c}$ of 146.3 °C, while Resin 2 had a $T_{c}$ of 148.1 °C, resulting in a 2.0 °C difference. The Resin 2 additive has a strong effect on non-isothermal cure at low cooling rates but is more pronounced at higher rates.
- The instantaneous rate of cure of the tested temperatures were measured. It was observed that as the temperature increased from 10 °C up to 170 °C, the rate of cure reduced, and the heat of reaction increased. The amount of heat released from the different samples were close to each other.
- For a constant applied temperature, the conversion rate behaved in a constant fashion and depended on the instantaneous rate of cure. The amount of added catalyst defined the total degree of cure and was based on the total activation energy. For the variable heating temperature, the curing cycle was controlled as desired and provided the desired amount of cure with a proper peak during the thermal cycle.

The simulation of the degree of cure not only matched the experimental data but it also helped in predicting the cure at other temperatures not determined experimentally. This GUI calculator will be a useful tool to determine the cure behaviors of resin systems and will save time and materials. The calculator is useful for different manufacturing processes to understand resin curing behavior and to optimize the processing conditions. Further study would analyze the localized rate of conversion and degree of cure of engineering parts, including thick composite plates with further modification of the numerical model.

## Figures and Tables

**Table 1:** DSC run time with respect to isothermal temperatures.

Temperature (°C)	DSC Run Time (min)
10–20	180
30–50	120
60–120	30
170	30

**Table 2:** TGA data for Resin 1 and Resin 2 at different heating temperatures.

Resin Type	$T_{5}$ (°C)	$T_{10}$ (°C)	$T_{max}$ (°C)
Resin 1	243.05	304.64	416.88
Resin 2	296.44	324.87	423.84

**Table 3:** Review of TGA for different UPR systems.

Literature Study	$T_{5}$ (°C)	$T_{10}$ (°C)	$T_{max}$ (°C)
Dai et al. [75]	-	348	419
Pączkowski et al. [56]	-	336	394
Tibiletti et al. [76]	287	-	427
Bai et al. [77]	307	-	433
Average value	297	342	418

**Table 4:** Parameters of the heating profile for the variable processing temperatures and degree of cure.

Temperature Profile	Ramp Up (°C/min) for 30 min	Dwell Time (min)	Ramp Down (°C/min) for 30 min	Maximum Calculated DOC (%)
1	0.15	30	0.10	63.19
2	0.25	30	0.25	66.05
3	0.75	30	0.50	73.02

## Data Availability

The original contributions presented in the study are included in the article, further inquiries can be directed to the corresponding author/s.
